# Epigallocatechin-3-gallate inhibits the growth of three-dimensional in vitro models of neuroblastoma cell SH-SY5Y

**DOI:** 10.1007/s11010-021-04154-w

**Published:** 2021-04-16

**Authors:** Xiao Wan, Wenbo Wang, Zhu Liang

**Affiliations:** 1 Target Discovery Institute, Nuffield Department of Medicine, University of Oxford, Oxford, England, UK; 2 Nuffield Department of Medicine Research Building, University of Oxford, Old Road Campus, Oxford, OX3 7FZ England, UK

**Keywords:** 3D in vitro models, DRYK1A, Vascular endothelial cells, Human umbilical vein endothelial cells, Neuroblastoma

## Abstract

The aim of the study is to investigate the potential of using three-dimensional (3D) in vitro neuroblastoma models to mimic the neuroblastoma microenvironment by testing a potential therapeutic compound—the natural extract epigallocatechin-3-gallate (EGCG), and to further elucidate the roles of DYRK1A in the growth and differentiation of neuroblastoma tissue. In vitro models based on a classic neuroblastoma cell line SH-SY5Y were employed, including 3D models with extracellular matrix and co-cultured with vascular endothelial cells. Cell viability was tested using AlamarBlue and Resazurin assay. The growth and differentiation of in vitro models of SH-SY5Y were analysed based on microscopy images obtained from immunofluorescence or real-time imaging. Protein expression level was investigated using immunoblotting analysis. The two-dimensional (2D) in vitro model implies the cytotoxicity and DYRK1A inhibition effect of EGCG and shows the induction of neuronal differentiation marker TuJ1. 3D in vitro models suggest that EGCG treatment compromised the growth of SH-SY5Y multicellular 3D spheroids and the viability of SH-SY5Y cultured in 3D Matrigel matrix. In addition, co-culture of SH-SY5Y with human vascular umbilical vein endothelial cells implied the inhibitory effects by EGCG in a vascularised microenvironment. In this study, novel 3D in vitro models of neuroblastoma were established in the application of testing a potential anti-cancer candidate compound EGCG. In pursuit of the goals of the 3Rs (replacement, reduction and refinement), the usage of these 3D in vitro models has the potential to reduce and eventually replace current animal models used in neuroblastoma research. The DYRK1A inhibiting nature of EGCG, together with the facts that EGCG inhibits the growth and induces the differentiation of neuroblastoma in vitro models, suggests an oncogene role of DRYK1A.

**Supporting Information:**

The online version of this article (10.1007/s11010-021-04154-w) contains supplementary material, which is available to authorized users.

## Introduction

Neuroblastoma is a rare neural crest-originated tumour, mainly found in young infants and children. Among the potential targets to intervene with this disease, DYRK1A, which is ubiquitously expressed during early brain development, emerged as a promising therapeutic candidate for unmet clinical needs [1]. Amongst DYRK family, DYRK1A is the most extensively studied kinase and has been associated with cancer and neurological diseases, such as Down’s syndrome (DS) and neurodegenerative diseases [2]. Under physiological condition, DYRK1A is implicated in neuron development as a negative regulator, halting the cell cycle progression at transition from G1/G0 to S phase [3]. The potential clinical significance of DYRK1A has stimulated considerable interests in seeking for effective and selective inhibitors [4]. Among the natural inhibitors for DYRK1A, green tea-derived epigallocatechin-3-gallate (EGCG) shows potent selective inhibition in both Down syndrome-related symptoms and tumourigenesis [5, 6], further clinical trials on EGCG would offer more empirical evidence and improve the understanding of its functional activity in cancer development [NCT02891538 (colorectal cancer), NCT02577393 (lung cancer) etc.]

To the best of our knowledge in the study of neuroblastoma, this rare tumour is limited by the availability of preclinical models, for which 2D in vitro models and animal models were mostly used [7]. There is a previous report on testing EGCG on neuroblastoma cell line SH-SY5Y, but in the context of neurotoxicity rather than investigating the direct effects of EGCG on this neuroblastoma cell line [8]. The aim of this study is to establish a range of 3D in vitro models using the neuroblastoma cell line SH-SY5Y, with complexity ranging from traditional 2D model, to a 3D model in Matrigel and to a co-culture model with angiogenesis factors. The application potential of these models will be tested and the potential of EGCG using these 3D in vitro models will be discussed.

## Methods

### Cell preparation

Neuroblastoma cell line SH-SY5Y (ATCC) was cultured in high-glucose Dulbecco’s Modified Eagle Medium (DMEM, Gibco, UK) supplemented with 10% (v/v) foetal bovine serum (Gibco, UK) and 100 U penicillin/ml and 100 lg streptomycin/mL (Gibco, UK). The human umbilical vein endothelial cells (HUVECs) were cultured in endothelial growth medium (EGM, Lonza, UK). All the cells were cultured in a humidified incubator at 37 °C with 5% CO_2_.

### 3D culture of SH-SY5Y and co-culture with HUVECs

**Fig. 1:**
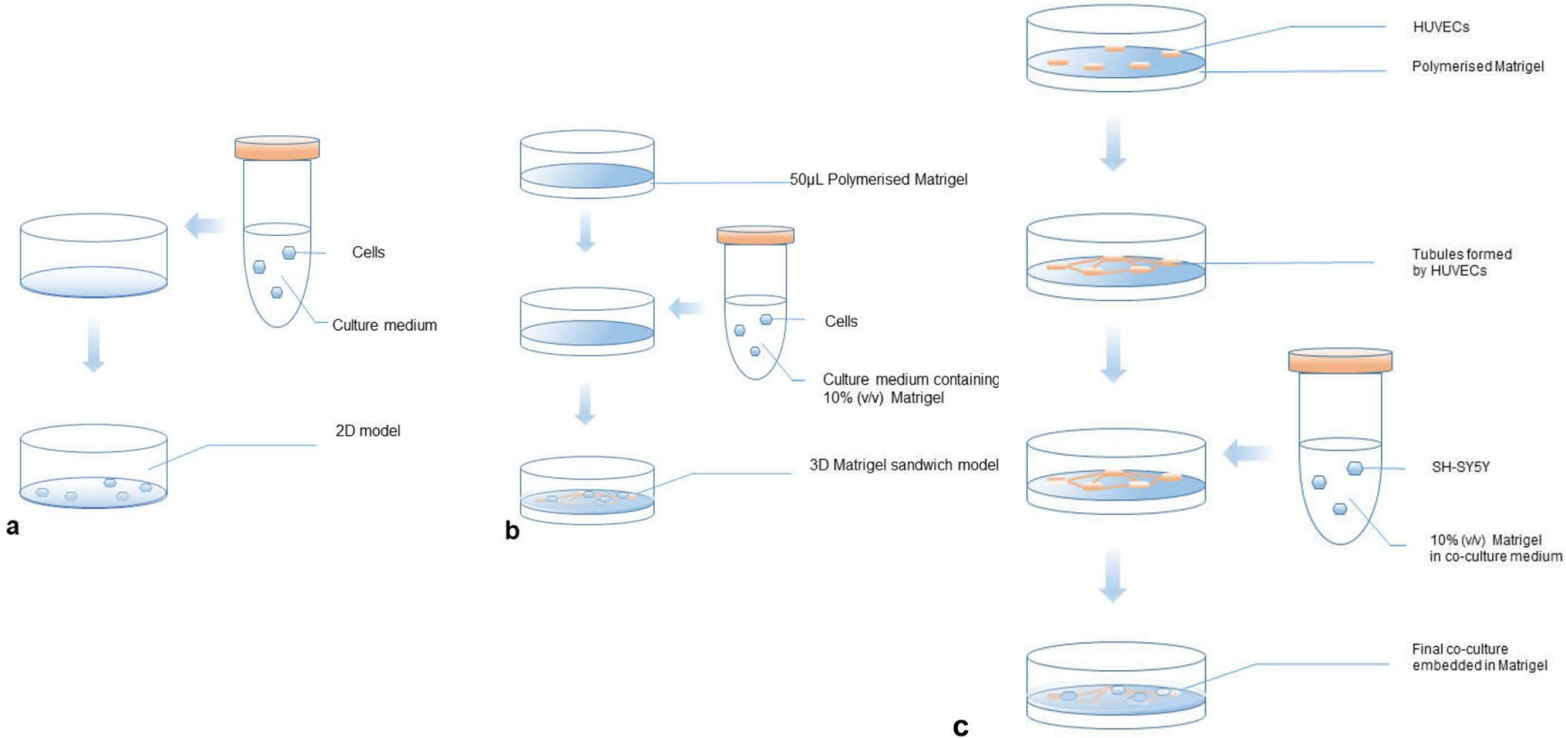
Schematic procedures for 2D and 3D in vitro models used in this study. **a** 2D monolayer culture of SH-SY5Y; **b** 3D Matrigel sandwich model of SH-SY5Y. **c** 3D Matrigel sandwich co-cultured with vascular endothelial cells HUVECs model of SH-SY5Y

3D multicellular spheroids were cultured based on a previous study [9]. A modified protocol was developed to form the Matrigel sandwich structure for 3D in vitro models, based on the previous reports from our lab [11] as shown in Fig. 1. Briefly, to form the bottom layer of Matrigel, 50 µL Matrigel (Sigma-Aldrich) was added into each well which had been pre-chilled on ice, and then the plates were incubated at 37 °C for 30 min, allowing the Matrigel to polymerise. SH-SY5Y cells in 10% Matrigel (v/v) in 500 µL pre-chilled EBM-2 supplemented with 2% FBS and 1% penicillin–streptomycin were added onto the polymerised Matrigel at the density of 3–5 × 10^4^ cells/mL. In the co-culture model, HUVECs were added together with SH-SY5Y onto the polymerised gel layer at the density of 1–1.5 × 10^5^ cells/mL in 500 µL endothelial basic medium (EBM-2, Lonza, UK) supplemented with 2% FBS (Life Technologies, US) and 1% penicillin–streptomycin (Thermo Fisher). The 3D in vitro systems were maintained at 37 °C, 5% CO_2_ for at least 24 h before further drug testing.

### CRISPR-Cas9 knock-out of DYRK1A as quality control

We obtained a CRISPR-Cas9 KO kit sample from Synthego (Switzerland) as a positive control for DYRK1A inhibition, in addition to the treatment of known inhibitors for DYRK1A including INDY (Tocri UK), Leucettin 41 (also known as L41, Cambridge Bioscience, UK), K04005 and K04179 (kindly provided by Professor Stefan Knapp, SGC University of Oxford). Briefly, the Cas9 and single-guided RNA (sgRNA) was separately prepared in serum-reduced medium Opti-MEM (Thermo Fisher), and then the RNP complex was assembled by incubation of Cas9 and sgRNA at room temperature for 10 min. The RNP complex was then added to the SH-SY5Y cells, and the cell lysates were collected and analysed by western blotting after 48 days. Four sgRNA sequences were designed by algorithm developed by Synthego, and the information is provided in supplementary data.

### Cell proliferation assay

Resazurin assay (AlamarBlue) kit was purchased from Abcam, UK and the assay was carried out based on the instructions. Briefly, the working solution was incubated with cells for 2.5 h. Emission at 590 nm with the excitation wavelength as 544 nm was read on a micro-plate reader (Omega, UK). Another cell viability assay, MTT assay, was used for time-lapse monitoring of cell viability in Fig. 5b. The kit was purchased from Sigma, and the absorbance was read at absorbance 490 nm after incubation with the reagent and treatment of DMSO based on the manual.

### Immunoblotting analysis

**Fig. 2:**
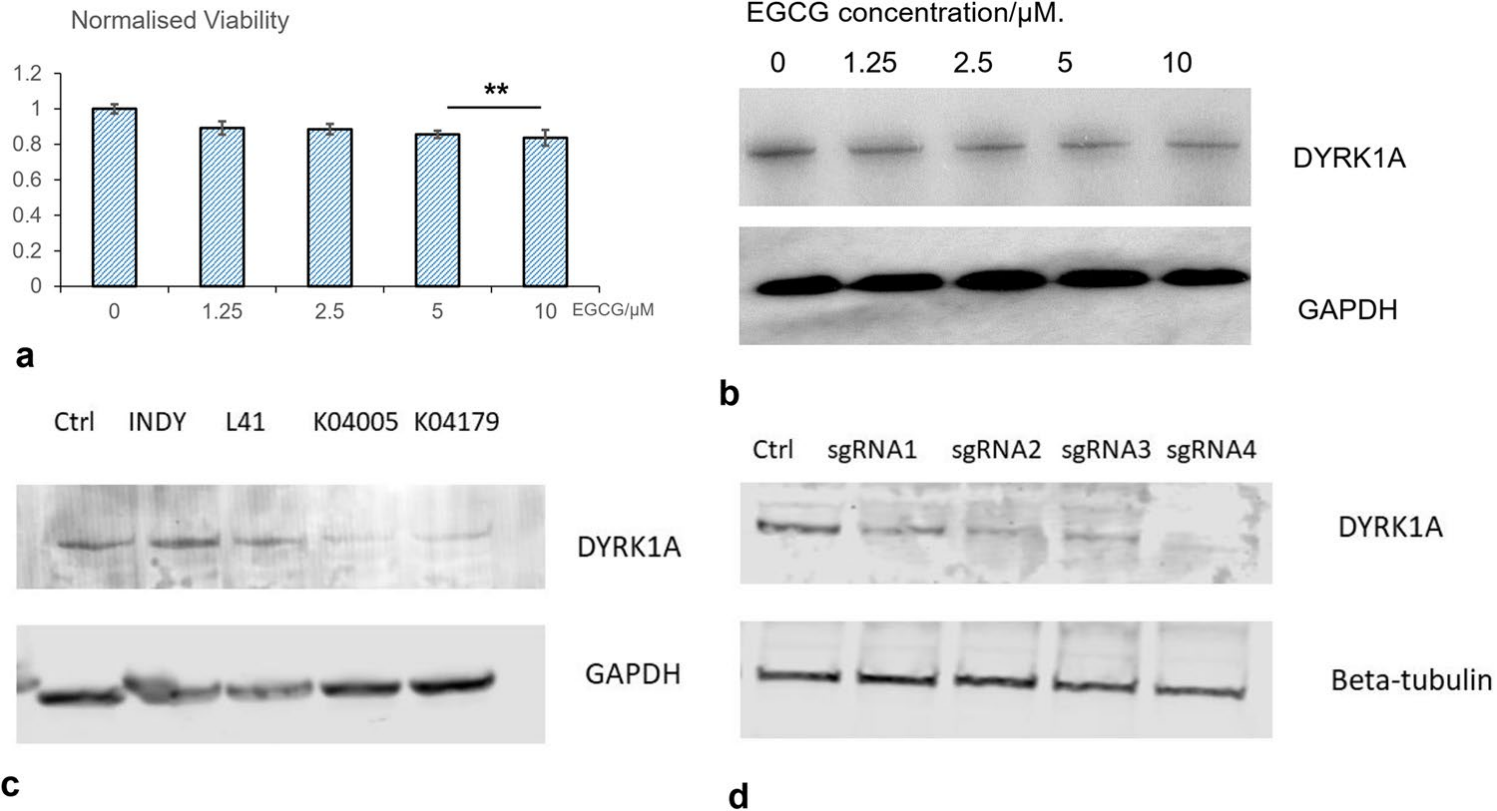
2D in vitro model implies the cytotoxicity and induction of neuronal differentiation DYRK1A inhibition effect of EGCG. **a** EGCG reduced the cell viability of SY5Y in a dosage-dependent manner. **b** Western blotting showed decrease of DYRK1A protein expression following the treatment with EGCG in a dosage-dependent manner. **c** Western blotting showed decrease of DYRK1A protein expression after SH-SY5Y cells were treated with known DYRK1A inhibitors. **d** CRISPR-Cas9 knock-out of DYRK1A using four-designed single-guided RNA (sgRNA) significantly decreased the protein level of DYRK1A

Cells were lysed on ice using RIPA lysis (ENZO) buffer-containing protease inhibitor cocktail (Thermo Fisher Halt™) and 1% benzonase to remove nuclei. The samples were then diluted in 5× Laemmli loading buffer. Proteins were then analysed in 4–12% Bis–Tris Nu-PAGE gels (Invitrogen) and transferred to PVDF membrane (Merck Millipore). After blocking in 5% milk (Sigma-Aldrich) at room temperature for 1.5 h, membranes were incubated overnight in blocking buffer-containing specific antibody (DYRK1A rabbit anti-human antibody, Santa Cruz). The following day, membranes were washed in PBST and then incubated with 5% milk containing the peroxide-conjugated anti-rabbit secondary antibody (Jackson Immunoresearch). Membranes were washed again, and bands were visualised using X-ray system (Kodak). For the images in Fig. 2c, d, the secondary antibody were labelled with fluorescence emissions at 680 nm (mouse) and 800 nm (rabbit) and images with a scanner (LI-COR).

### 3D culture whole fixation Immunofluorescence

For immunofluorescence, cells in 3D culture were stained using a protocol modified for sandwich Matrigel [11, 20]. The samples were first rinsed by PBS—glycine (100 mM glycine in PBS) three times, then blocked with 10% goat serum (Sigma), 1% goat F(ab′)2 anti-mouse immunoglobulin G (Caltag, UK) in staining buffer (PBS supplemented with 0.2% TritonX-100, 0.1% BSA, and 0.05% Tween 20). The samples were then incubated with the desired primary antibodies at 4 °C overnight (Tuj1 rabbit anti-human (Abcam, UK), SOX-2 mouse anti-human (Merck Millipore, UK)), followed by the incubation in the suitable second antibodies (Goat anti-rabbit Alexa569, goat anti-mouse Alexa488 from ThermoFisher, UK) at room temperature for 45 min at least and finally were mounted with diaminophenylindole (DAPI) mounting medium (Abcam, UK). Images were taken on a confocal microscope (Zeiss 710), viewed in ZEN software (Zeiss) and exported into ImageJ (http://imagej.nih.gov/ij/) for processing.

### Image processing

All the exported TIF images were segmented and analysed in ImageJ. Briefly, exported images were transformed into 8-bit image types. The best contrast was obtained using a Z-stack function of NIS Viewer (Nikon) to make that the areas covered by cells are brighter than the background for the best segmentation output. Then the images were segmented by function ‘ImageThreshold’. Particle analysis was used to measure all the segmented areas and the areas were added up in each group. For the fluorescence intensity measurement, the areas were selected manually by multi-point selection tool and the function ‘Measure’ was used for each cell. Figure s1 in the supplementary data demonstrates the segmentation process. For 3D multicellular spheroids, as the objects are darker than the background, the ‘dark background’ must be un-checked in the threshold function for the dark areas to be selected and analysed.

### Statistics

Besides specific instructions, all experiments were repeated three times independently. Two-tailed unpaired *t* test was used to compare the significant difference between different culture methods. *p* < 0.05 was considered significantly different.

## Results

### 2D in vitro model implies the cytotoxicity and induction of neuronal differentiation of EGCG

**Fig. 3:**
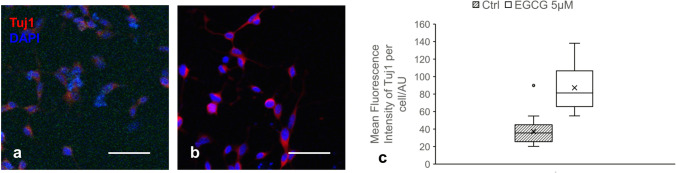
EGCG promoted differentiation marker Tuj1 of in vitro neuroblastoma model. Representative microscopy images of SH-SY5Y cell line treated with **a **vehicle control or **b** 5 µM EGCG for 72 h and stained with fluorescent probe for Tuj1 (red) and nuclei (DAPI, blue); **c** Quantification of the fluorescence intensity showing significant difference between the control group and the group treated by 5 µM EGCG (*n* = 20). Based on Students’ *t* test, *p* < 0.05. (Color figure online)

A dose-dependent inhibition on cell viability by treatment of EGCG after 24 h was observed in SY5Y cells cultured in 2D cell monolayer, for the dosage range 1.25 µM to 10 µM. As shown in Fig. 2a, the viability of SH-SY5Y cultured in 2D monolayer significantly decreased after 24 h treatment with 2.5 µM EGCG (*p* = 0.032). This cytotoxic effect of EGCG is even more significant when the concentration of EGCG is increased to 5 µM (*p* = 0.00028). Consistent with previous studies, we observed that EGCG significantly inhibited the expression of DYRK1A protein in a dose-dependent manner (Fig. 2b). Known DYRK1A inhibitors INDY, Leucettin 41, and two selective DYRK1A inhibitors synthesized by our collaborators were used as positive control for Western Blot as shown in Fig. 2c. We also did a CRISPR knock-out experiment, and the Western Blot showed that DYRK1A expression was significantly reduced after 48 h of treatment by four-designed single-guide RNA (sgRNA), as shown in Fig. 2d. Interestingly, the inhibitory effect of EGCG is accompanied by the increase of Tuj1 (class III β-tubulin), a widely used marker for early neuronal differentiation, as shown in Fig. 3a. After treatment with EGCG at the concentration of 5 µM for 72 h, the fluorescence intensity of Tuj1, labelled by immunofluorescence staining, increased significantly (*p* = 7.68412E−09), as plotted in Fig. 3b (*n* = 20).

### EGCG treatment compromised the growth of SH-SY5Y 3D spheroid and 3D Matrigel sandwich in vitro models

The SH-SY5Y multicellular spheroids without extracellular matrix which were treated by 5 µM EGCG showed a significant smaller size compared with the control group, with the average segmented areas of the spheroids as 291,544 µm^2^ compared with the 426,021 µm^2^ of the control groups. Increasing the concentration of EGCG to 10 µM caused the average spheroid area to a smaller size, 156,938 µm^2^ (Supplementary Fig. 2).

**Fig. 4:**
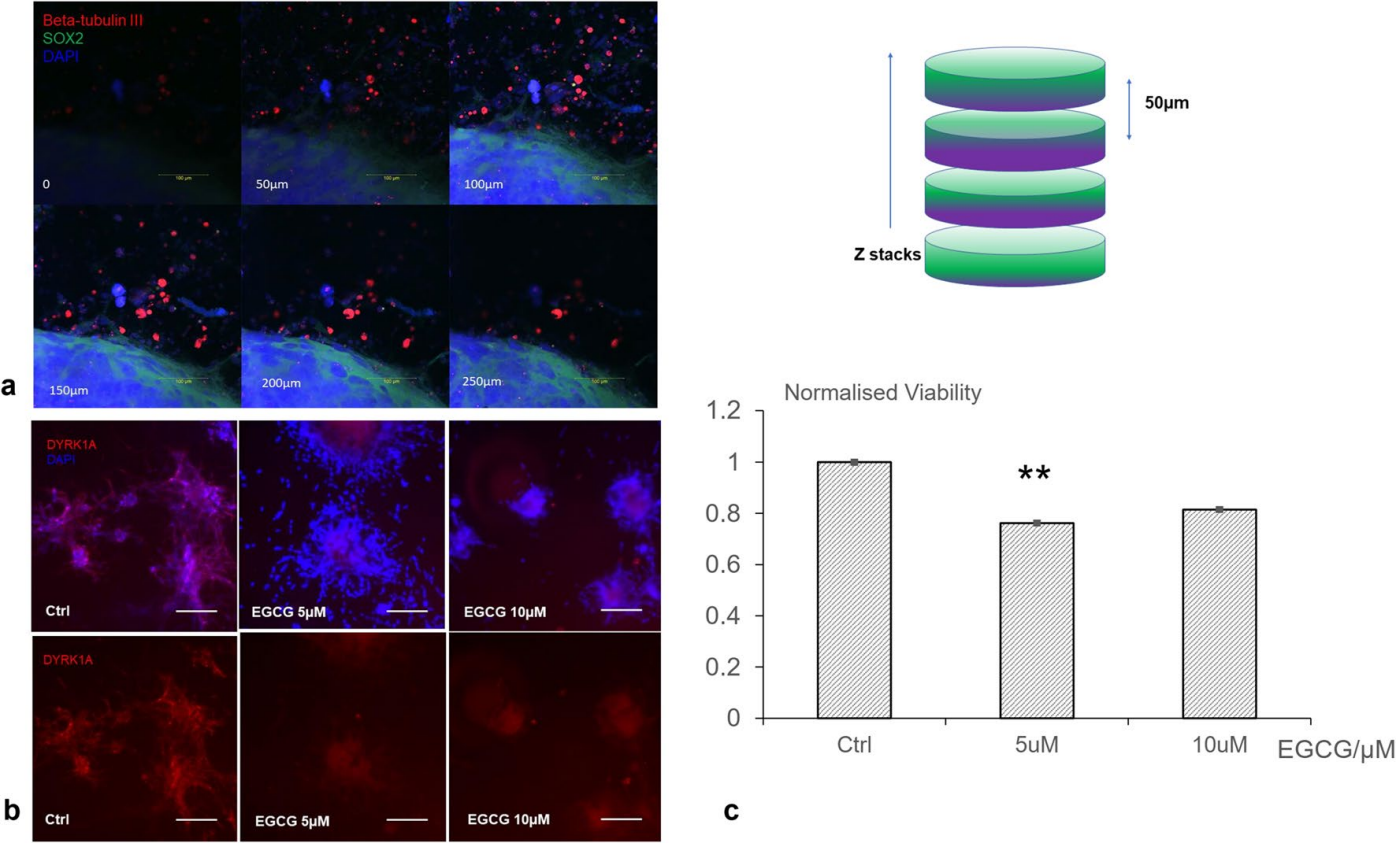
EGCG treatment reduced the viability of SH-SY5Y 3D Matrigel sandwich in vitro model. **a** Whole culture fixation and immunofluorescence imaging showing the culture had a thickness of 200 µm with Beta-tubulin III-positive stained neurite-like structures growing from the culture (red fluorescence). **b** Representative microscopy images of SH-SY5Y cultured in Matrigel sandwich suggested that EGCG impaired DYRK1A expression (red fluorescence) in Matrigel 3D-cultured neuroblastoma. **c** Viability quantification showed significant difference between the control group and the group treated by 5 µM EGCG (*n* = 4). The experiments were repeated three times. Based on Students’ *t* test, **p < 0.01. (Color figure online)

For the 3D sandwich culture of SH-SY5Y, we first characterised the model using the pluripotency marker SOX2 and early neuron differentiation marker Tuj1 (class III β-tubulin). Figure 4a shows the culture had a three-dimensional structure extending to 200 µm. It was also noticeable that the neurite-like protrusion growing from the 3D culture was positively stained with Tuj1. To investigate the effect of DYRK1A inhibition in a tumour microenvironment with biological relevance, we measured the effects of EGCG on the viability of 3D structures formed by SY5Y in a Matrigel-based 3D sandwich culture system. The 3D culture in Matrigel labelled with DYRK1 antibody by fluorescence labelling suggested a reduced fluorescence intensity caused by EGCG treatment (Fig. 4b), supporting the results in Fig. 3b. Treatment of EGCG 5 µM for 72 h caused a 24% percentage of viability reduction (Fig. 4c). Interestingly, increasing the concentration of EGCG to 10 µM did not cause a higher viability reduction.

### Co-culture of neuroblastoma cell SH-SY5Y with vascular cells human umbilical vein endothelial cells (HUVECs) suggested inhibition effects by EGCG in a vascularised microenvironment

**Fig. 5:**
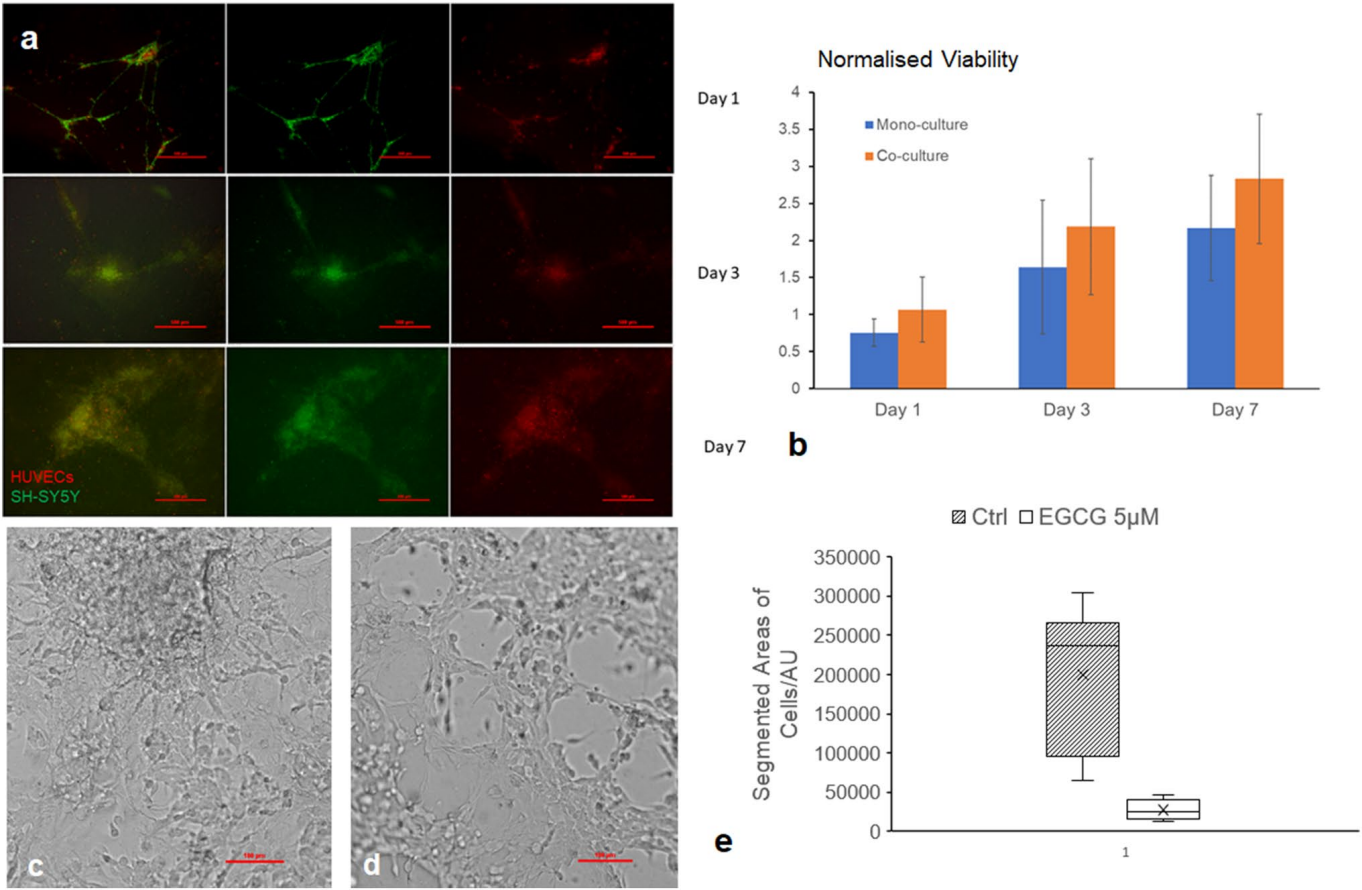
Characterisation of 3D Co-culture Model of SH-SY5Y with HUVECs and EGCG significantly decreased the area growth of SH-SY5Y co-cultured with HUVECs in Matrigel sandwich after 24 h treatment. **a** Living cell imaging with HUVECs stained by CellTracker Orange and SH-SY5Y labelled by CellTracker Green showing the maintenance of co-culture over time. **b** MTT proliferation assay showing the viability of co-culture as well as mono-culture of SH-SY5Y in Matrigel sandwich stably increased over 7 days of culture. **c** 3D Ctrl; **d** EGCG-treated 3D for 24 h. Scale bar: 100 µm. **e** Areas covered by co-culture quantified by ImageJ segmentation function and visualised by Box and Wisker plot. *n* = 5. Based on Students’ *t* test, *p* < 0.05

SH-SY5Y and HUVECs were co-cultured in 3D sandwich Matrigel, and the morphology and viability were characterised in Fig. 5a, b respectively. The cells kept proliferating and maintained the 3D structure indicated by increased viability and maintained co-culture structure. As shown in Fig. 5a, c, SH-SY5Y co-cultured with HUVECs were seeded at the initial seeding ratio 1:3 and formed a stable 3D structure in the 3D in vitro models. Further quantification in Fig. 5e showed that EGCG at a concentration of 5 µM significantly decreased the areas covered by co-culture of SH-SY5Y with HUVECs, with the p value as 0.0175 based on Student’s *t* test (*n* = 6). Intriguingly, when cultured in 2D monolayer, the co-culture of SH-SY5Y and HUVECs did not show significant difference, with the overall area of cells of EGCG-treated group even slightly larger than the control group (Fig. s3).

## Discussion

Previously researchers have reported the application of in vitro models with 3D features, such as spheroids formed by neuroblastoma cell line [10]. In those reports, mostly low-attachment plates were used, in the absence of extracellular matrix. To investigate the effect of drugs in a tumour microenvironment with biological relevance, we used a model formed by cancer cells in a Matrigel-based 3D sandwich culture system, which was developed previously by Wan et al. [11]. Here, we present a 3D model integrating extracellular matrix mimicked by Matrigel, a widely applied matrix replacement. We noticed that 3D Matrigel-based neuroblastoma model shows the influence of DYRK1A inhibition on  the 3D culture growth. EGCG inhibited DYRK1A in Matrigel 2D and 3D-cultured neuroblastoma cell SH-SY5Y. Integration of vascular endothelial cells HUVECs in this 3D sandwich model further showed the inhibitory effect of EGCG after 24 h of treatment compared with control group regarding to the segmented areas of the co-culture. Previous clinical observations suggest the interference of ABT-751 on tubulin polymerisation and its antitumour effects [12]; together with our results, it suggests that interfering with the cytoskeleton, particularly tubulin, could be a potential direction for future antitumour research.

SH-SY5Y has been widely used as a cell model. Compared with primary cells, these cells are easy to grow with relatively low cost [13]. It is also interesting that other researchers reported the protection of EGCG on SH-SY5Y in a different context [14]. The suppression of differentiation was observed on SH-SY5Y pre-treated with EGCG, attenuating the cytotoxicity induced by 6-hydroxydopamine [14]. In this study, SH-SY5Y was used as a neurogenesis model rather than a neuroblastoma cell line, and the treatment regime of EGCG is different. Among many reports showing the neuroprotection effects of EGCG, one report also proposed the potential of EGCG penetrating blood-brain-barrier (BBB) and suggested the pro-proliferation effect of EGCG at a low concentration (0.1 µM) [15]. Of particular note, the highest concentration of EGCG used for pro-proliferative function was 1 µM, which is lower than the lowest concentration used in our study. This suggests the dose-dependent manner of EGCG on SH-SY5Y, and a higher concentration might be needed for chemoprevention application of EGCG.

While DYRK1A has been proven to be an essential regulator in normal brain development and neurodegenerative diseases, the gaps still exist in our knowledge regarding its functional activities in tumourigenesis. Previous studies have suggested both oncogenic and tumour-suppressive roles for DYRK1A in cancer development and progression [16]. In this study, we found the DYRK1A inhibitor EGCG impaired neuroblastoma growth, suggesting that DYRK1A could be a therapeutic target and EGCG is a potential anti-cancer drug. We recognised that the clear mechanism of EGCG acting on tumours either DYRK1A dependent or involving other pathways worth further studying. We recently had a pre-application enquiry with Neuroblastoma UK and the initial comments were ‘exciting, opening new avenues for therapy’ so we are looking forward to studying this further if future funding is secured. It was also suggested by other researchers that the anti-cancer activities of EGCG and green tea might not be limited to DYRK1A inhibition, but their proven safety over the history makes them good drug candidates [17].

Neuroblastoma is characterised by a high level of vascularisation [18]. Previous reports have demonstrated the anti-angiogenesis effects of DYRK1A inhibitors [19]. In this study, to mimic this feature with in vitro models, vascular endothelial cells HUVECs, which are widely used for angiogenesis assay, were co-cultured with SH-SY5Y. In addition, both cells were studied in the absence and presence of EGCG separately. A range of parameters, including cell viability, neuronal differentiation marker Tuj1, and segmented area of cells were measured and compared between 2 and 3D in vitro models of either HUVECs or SH-SY5Y co-culture model or mono-culture of SH-SY5Y. In the 3D model, EGCG decreased the tumour cell area compared with the control group. Interestingly, co-culture in 2D monolayer did not show a difference. It was observed before that certain compounds would not show efficacy in 2D culture. This could be caused by the involvement of a hypoxia-related mechanism of the tested compound, which can only be revealed in the 3D structures formed by 3D in vitro models such as multicellular spheroids [20].

In conclusion, in this study (1) 3D in vitro models of neuroblastoma were established. (2) A potential anti-cancer candidate compound EGCG was tested by applying these models. (3) The usage of these 3D in vitro models of neuroblastoma has 3Rs significance to reduce and eventually replace current animal models used in neuroblastoma research.

## Supplementary Information

Below is the link to the electronic supplementary material.

Supplementary file1 (DOCX 2559 KB)

## Data Availability

The original data will be provided if required.
