# Supplementary material for: Epigallocatechin-3-gallate inhibits the growth of three-dimensional in vitro models of neuroblastoma cell SH-SY5Y
Source: Mol Cell Biochem. 2021 Apr 16;476(8):3141–8. doi: 10.1007/s11010-021-04154-w (PMC8263418; doi:10.1007/s11010-021-04154-w)
Supplement: Supplementary file 1 — Supplementary file1 (DOCX 2559 KB) [file 11010_2021_4154_MOESM1_ESM.docx]

**Epigallocatechin-3-Gallate Inhibits the Growth of Three-dimensional *In Vitro* Models of Neuroblastoma Cell SH-SY5Y**

Xiao Wan*, Wenbo Wang, Zhu Liang

Target Discovery Institute, Nuffield Department of Medicine, University of Oxford

**Correspondence**

[xiao.wan@ndm.ox.ac.uk](mailto:xiao.wan@ndm.ox.ac.uk)

Nuffield Department of Medicine Research Building, University of Oxford Old Road Campus, OX3 7FZ

Supplementary Data


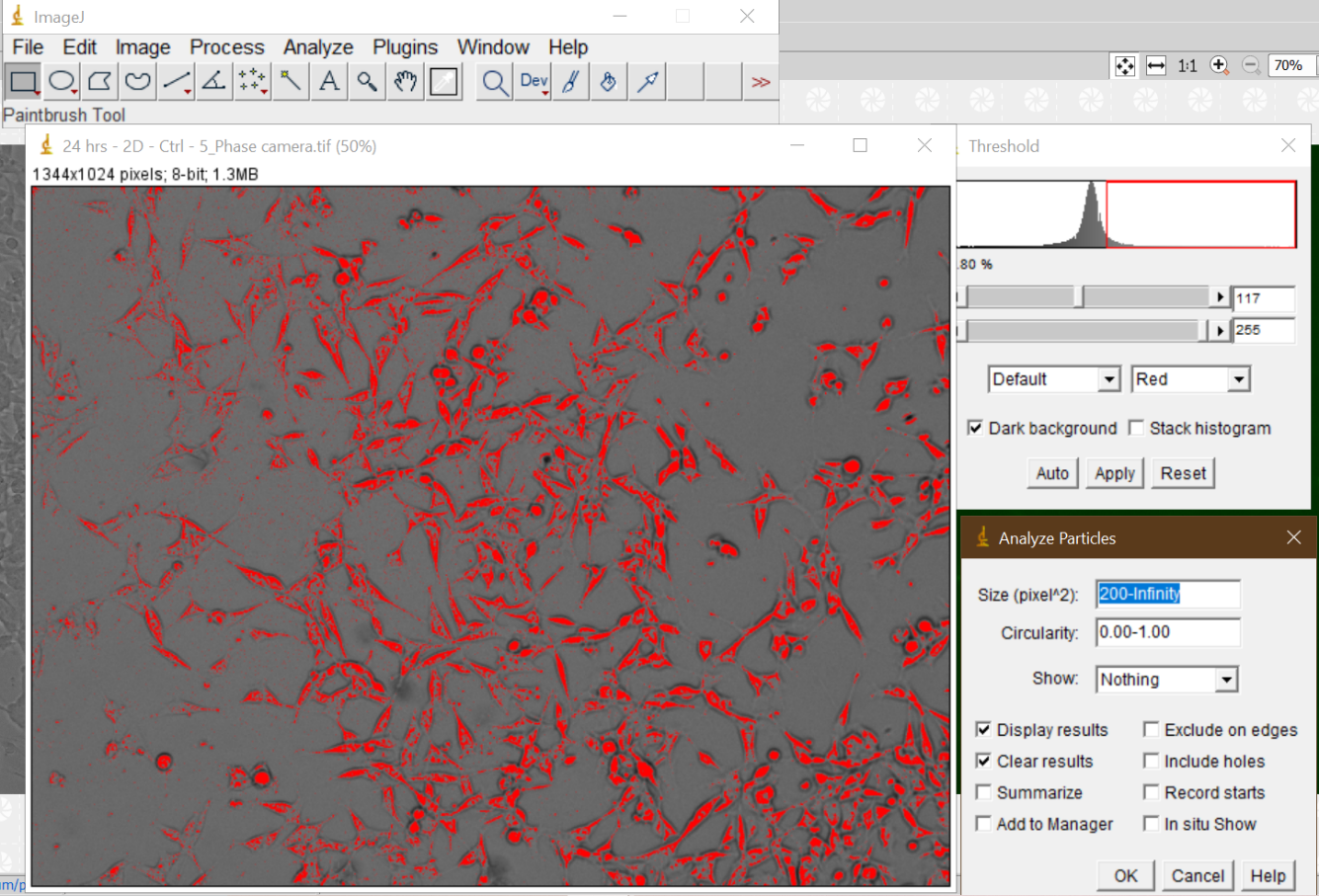
(a)


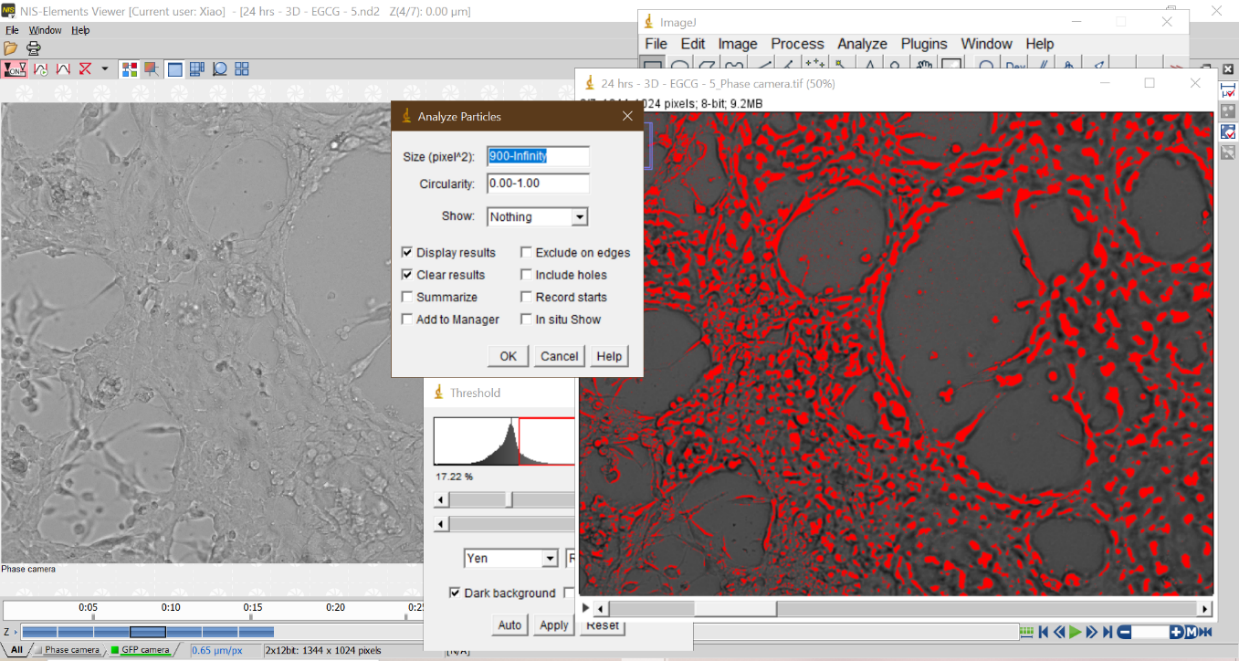
(b)

Figure s1. Image analysis of 2D culture of SH-SY5Y using ImageJ. (a) Segmentation of 2D culture of SH-SY5Y; (b) Segmentation of 3D culture of SH-SY5Y.


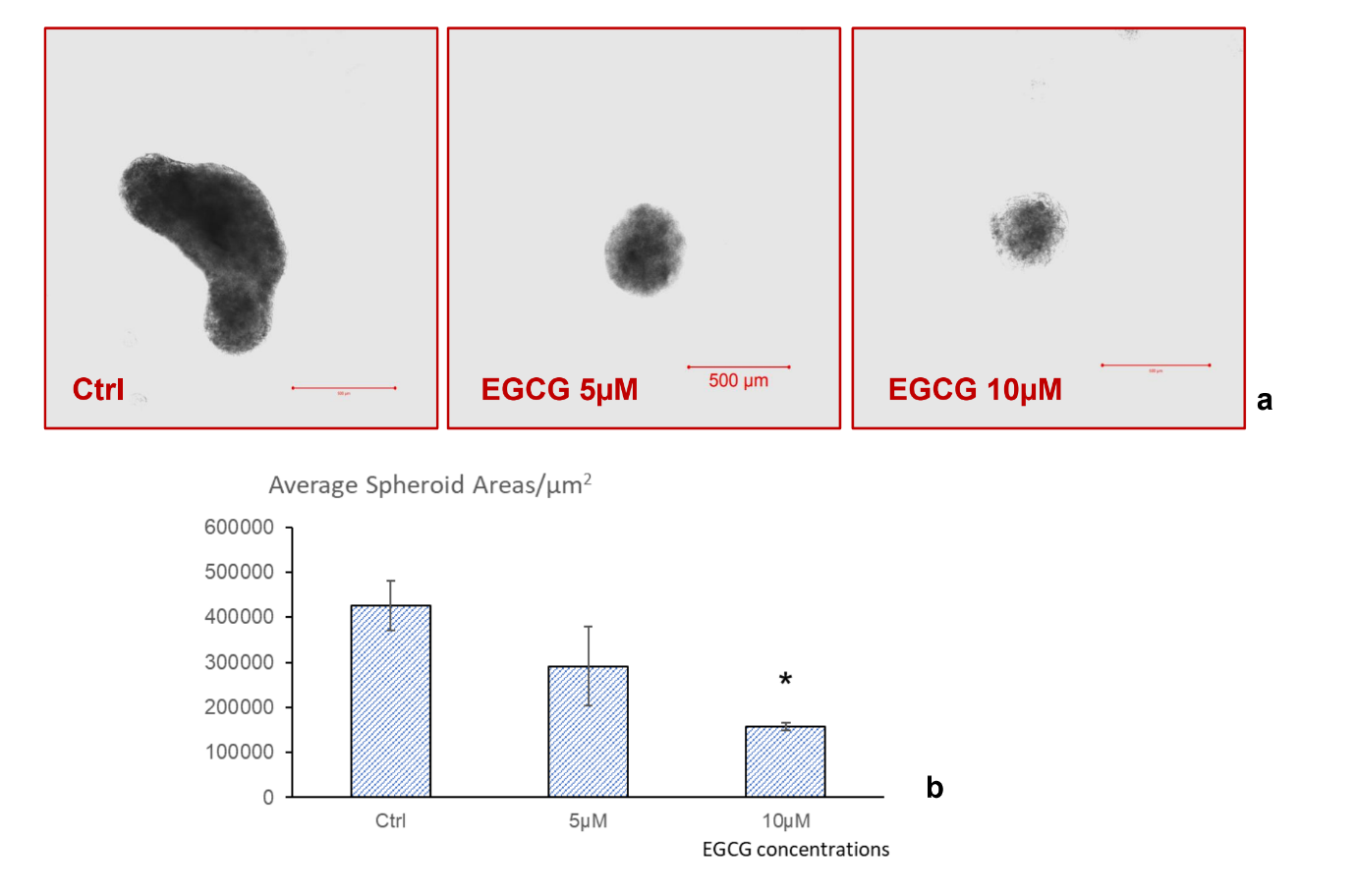


**Figure s2. EGCG treatment compromised the growth of SH-SY5Y 3D spheroid.** (a) EGCG impaired the growth of classic multicellular spheroids models of SH-SY5Y (b) Spheroid size quantification (n=3). *p < 0.05 based Student’s T-Test.


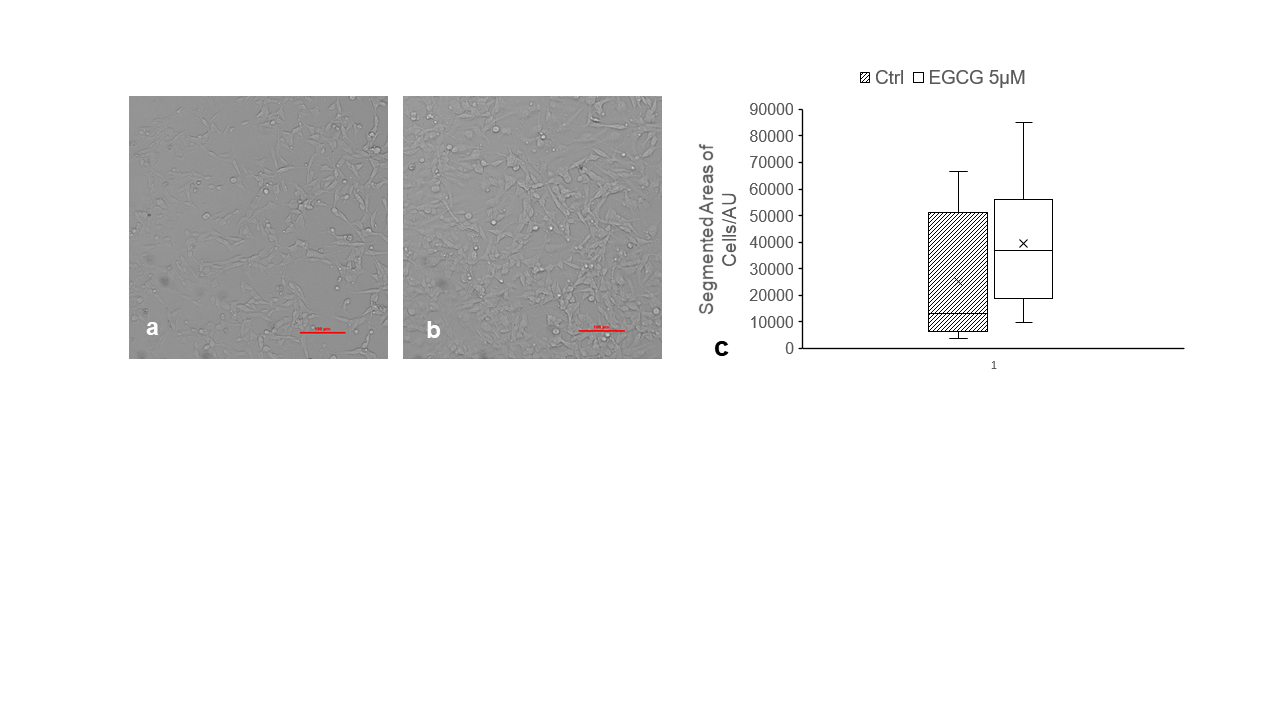


Figure s3. 2D co-culture did not shown significant difference after EGCG treatment, compared with results shown in Figure 5. (a) 2D Ctrl; (b) EGCG treated 2D for 24 hours; (c) No significant difference between the cell culture areas of 2D mono-culture of control group and EGCG treated group (5µM for 24 hours). Box and wisker plot of the data (n=6).
